# Fine mapping and candidate gene analysis of a dravet syndrome modifier locus on mouse chromosome 11

**DOI:** 10.1007/s00335-022-09955-y

**Published:** 2022-05-23

**Authors:** Jennifer A. Kearney, Letonia D. Copeland-Hardin, Samantha Duarte, Nicole A. Zachwieja, Isaiah K. Eckart-Frank, Nicole A. Hawkins

**Affiliations:** 1 Department of Pharmacology, Northwestern University Feinberg School of Medicine, 320 E. Superior St., Searle 8-510, Chicago, IL 60611 USA; 2 Driskill Graduate Program in Life Sciences, Northwestern University Feinberg School of Medicine, Chicago, IL 60611 USA

## Abstract

Pathogenic variants in *SCN1A* result in a spectrum of phenotypes ranging from mild febrile seizures to Dravet syndrome, a severe infant-onset epileptic encephalopathy. Individuals with Dravet syndrome have developmental delays, elevated risk for sudden unexpected death in epilepsy (SUDEP), and have multiple seizure types that are often refractory to treatment. Although most Dravet syndrome variants arise de novo, there are cases where an *SCN1A* variant was inherited from mildly affected parents, as well as some individuals with de novo loss-of-function or truncation mutations that presented with milder phenotypes. This suggests that disease severity is influenced by other factors that modify expressivity of the primary mutation, which likely includes genetic modifiers. Consistent with this, the *Scn1a*^+/−^ mouse model of Dravet syndrome exhibits strain-dependent variable phenotype severity. *Scn1a*^+/−^ mice on the 129S6/SvEvTac (129) strain have no overt phenotype and a normal lifespan, while [C57BL/6Jx129]F1.*Scn1a*^+/−^ mice have severe epilepsy with high rates of premature death. Low resolution genetic mapping identified several Dravet syndrome modifier (*Dsm*) loci responsible for the strain-dependent difference in survival of *Scn1a*^+/−^ mice. To confirm the *Dsm5* locus and refine its position, we generated interval-specific congenic strains carrying 129-derived chromosome 11 alleles on the C57BL/6J strain and localized *Dsm5* to a 5.9 Mb minimal region. We then performed candidate gene analysis in the modifier region. Consideration of brain-expressed genes with expression or coding sequence differences between strains along with gene function suggested numerous strong candidates, including several protein coding genes and two miRNAs that may regulate *Scn1a* transcript.

## Introduction

Dravet syndrome is an infant-onset epileptic encephalopathy caused by haploinsufficiency for *SCN1A*. Seizure onset usually occurs between 6 and 18 months of age with generalized tonic–clonic or hemiclonic seizures, often precipitated by fever. Individuals subsequently develop pleiomorphic afebrile seizure types that often respond poorly to conventional therapies (Dravet 2011; Dravet and Oguni 2013; Wirrell et al. 2017). Development is normal prior to onset, but disease progression is frequently accompanied by stagnation or decline of psychomotor and cognitive development (Battaglia et al. 2021; Dravet 2011). Individuals with Dravet syndrome have a significantly increased risk of mortality, mainly attributed to prolonged status epilepticus in early childhood and sudden unexplained death in epilepsy (SUDEP) in adolescents and adults (Dravet 2011). In most cases, the *SCN1A* variants arise de novo and result in heterozygous loss-of-function (Li et al. 2021). However, there have been reports of Dravet syndrome caused by *SCN1A* variants inherited from mildly affected parents, as well as some individuals with de novo loss-of-function or premature truncation variants that presented with milder phenotypes, like generalized epilepsy with febrile seizures plus (GEFS^+^) (Depienne et al. 2010, 2009; Goldberg-Stern et al. 2014; Guerrini et al. 2010; Nabbout et al. 2003; Osaka et al. 2007; Yordanova et al. 2011; Yu et al. 2010). This variable expressivity suggests that disease severity is influenced by additional factors, which may include genetic modifiers (de Lange et al. 2020; Hammer et al. 2017).

Mice with heterozygous targeted deletion of *Scn1a* (*Scn1a*^+/−^) are a well-validated model and recapitulate core features of Dravet syndrome, including epilepsy and sudden unexpected death following a seizure in otherwise healthy animals (SUDEP-like) (Kalume et al. 2013; Miller et al. 2014; Yu et al. 2006). The highest incidence of SUDEP-like deaths occurs in the 4th postnatal week and survival stabilizes after 6 weeks of age (Kalume et al. 2013; Miller et al. 2014). Genetic background dramatically influences phenotype severity and survival of *Scn1a*^+/−^ mice (Miller et al. 2014; Rubinstein et al. 2015; Yu et al. 2006). On the 129S6/SvEvTac (129) strain, there is no overt phenotype and 129.*Scn1a*^+/−^ mice live a normal lifespan. However, when 129.*Scn1a*^+/−^ mice are crossed with C57BL/6J (B6), the resulting [129xB6]F1.*Scn1a*^+/−^ mice (F1.*Scn1a*^+/−^) have a severe phenotype with spontaneous seizures and premature lethality (Miller et al. 2014). We previously performed genetic mapping and identified several Dravet syndrome modifier (*Dsm*) loci that influence the strain-dependent difference in survival (Miller et al. 2014).

In this study, we used interval-specific congenic (ISC) strains to fine map the *Dsm5* locus on chromosome 11 and identified a minimal interval that influenced survival, particularly in female *Scn1a*^+/−^ mice. Within the mapped interval, we performed an initial survey of potential candidate modifier genes and identified several high-priority candidate modifier genes.

## Methods

### Mice (NCBI Taxon ID 10090)

*Scn1a*^*tm1Kea*^ mice (MMRRC 037107-JAX) were generated by homologous recombination in TL1 ES cells (129S6/SvEvTac) as previously described (Miller et al. 2014). The line has been maintained as isogenic on 129S6/SvEvTac (129) by continuous backcrossing of 129.*Scn1a*^+/−^ heterozygotes to 129 inbred mice (129SVE, Taconic Biosciences, Germantown, NY, USA). *Trpv1* knockout mice were obtained from the Jackson Laboratory (Bar Harbor, ME, USA; RRID:IMSR_JAX:003,770). C57BL/6J mice were obtained from the Jackson Laboratory (Bar Harbor, ME, USA; RRID:IMSR_JAX:000664).

Mice were maintained in a Specific Pathogen Free (SPF) barrier facility with a 14:10 light:dark cycle and ad libitum access to food and water. All animal care and experimental procedures were approved by the Northwestern University Animal Care and Use Committee in accordance with the National Institutes of Health Guide for the Care and Use of Laboratory Animals. The principles outlined in the ARRIVE (Animal Research: Reporting of in vivo Experiments) guideline were considered when planning experiments (Percie du Sert et al. 2020).

### Interval specific congenic (ISC) lines

We generated nine ISC lines carrying 129-derived alleles on chromosome 11 on a C57BL/6 J (B6) background, designated as B6.129-Dsm5A through B6.129-Dsm5F. F1 progeny were generated by crossing 129 males with B6 females, and then successively crossed to B6 to generate congenic lines. Genotyping for chromosome 11 markers was performed at each generation and mice retaining 129 alleles were propagated. Whole genome and selective genotyping was performed at generations N2 and N5 to select breeders with low percentages of 129 in the rest of the genome. All lines were crossed to B6 for ≥ N9 generations prior to any experiments.

### Genotyping

DNA was prepared from tail biopsies (Gentra Puregene Mouse Tail Kit, Qiagen, Valencia, CA, USA). *Scn1a* genotype was determined by multiplex PCR using a common primer (5’- AGTCTGTACCAGGCAGAACTTG) and two allele specific primers (WT: 5’-CCCTGAGATGTGGGTGAATAG; KO: 5’‐AGACTGCCTTGGGAAAAGCG). Amplicons include a 357 bp WT product and a 200 bp KO product. Genotyping of microsatellite markers was performed by analysis of PCR products on 7% denaturing polyacrylamide gels stained with ethidium bromide. ISC breakpoints were refined using the mini Mouse Universal Genotyping Array (miniMUGA) (Neogen, Lincoln, NE, USA). Strain background was surveyed for *Trpv1* mice by miniMUGA genotyping (Neogen).

### Phenotyping

B6.129-*Dsm5* females were bred with heterozygous 129.*Scn1a*^+/−^ males to generate F1 offspring carrying homozygous 129/129 alleles or heterozygous 129/B6 alleles in *Dsm5*. Offspring were ear-tagged and genotyped at P12-14. At P19-21, mice were weaned into standard vivarium cages containing 4–5 mice of the same sex and age. Wild-type littermates were included in holding cages. Survival was monitored to 8 weeks of age. Over the 8-week period, mice were monitored daily for general health and any visibly unhealthy mouse (e.g., underweight, dehydrated, poorly groomed, or immobile) was euthanized and excluded from the study; this occurred rarely. The focus of the study was sudden and unexpected death in otherwise healthy appearing *Scn1a*^+/−^ mice. Survival was compared between groups using Kaplan–Meier analysis with p-values determined by LogRank Mantel–Cox tests. Group sizes were based on data simulations using data from our prior survival studies.

### Candidate gene analysis

We defined the *Dsm5* gene set using the Mouse GRCm38.p6 reference genome (B6) in Ensembl BioMart, which included classification by gene type. To define a subset of genes with CNS expression, we used the MGI database and EMBL-EBI Expression Atlas. Differential expression of *Dsm5* genes between 129 and B6 or F1 was assessed using two RNA-Seq datasets that we previously reported: (1) B6 and 129 forebrain (Hawkins et al. 2016); and (2) F1 and 129 hippocampus (Hawkins et al. 2019) (NCBI GEO GSE112627). Differential expression of miRNAs was assessed using a previously reported dataset (Trontti et al. 2018). To identify consequential coding sequence changes between the strains, we performed whole genome re-sequencing of 129S6/SvEvTac and compared with the C57BL/6J reference sequence and mouse strains in Ensembl (Mouse GRCm38.p6) (NCBI SRA PRJNA817075). The effect of single-nucleotide variants (SNVs) was assessed using the Ensembl Variant Effect Predictor tool (VEP) (McLaren et al. 2016), and SNVs classified as deleterious were retained. TargetScan and LncRRIsearch were used to predict targets of *Dsm5* high confidence miRNAs and lncRNAs, respectively (Agarwal et al. 2015; Fukunaga et al. 2019).

## Results

### Fine mapping of *Dsm5* to central chromosome 11

**Fig. 1:**
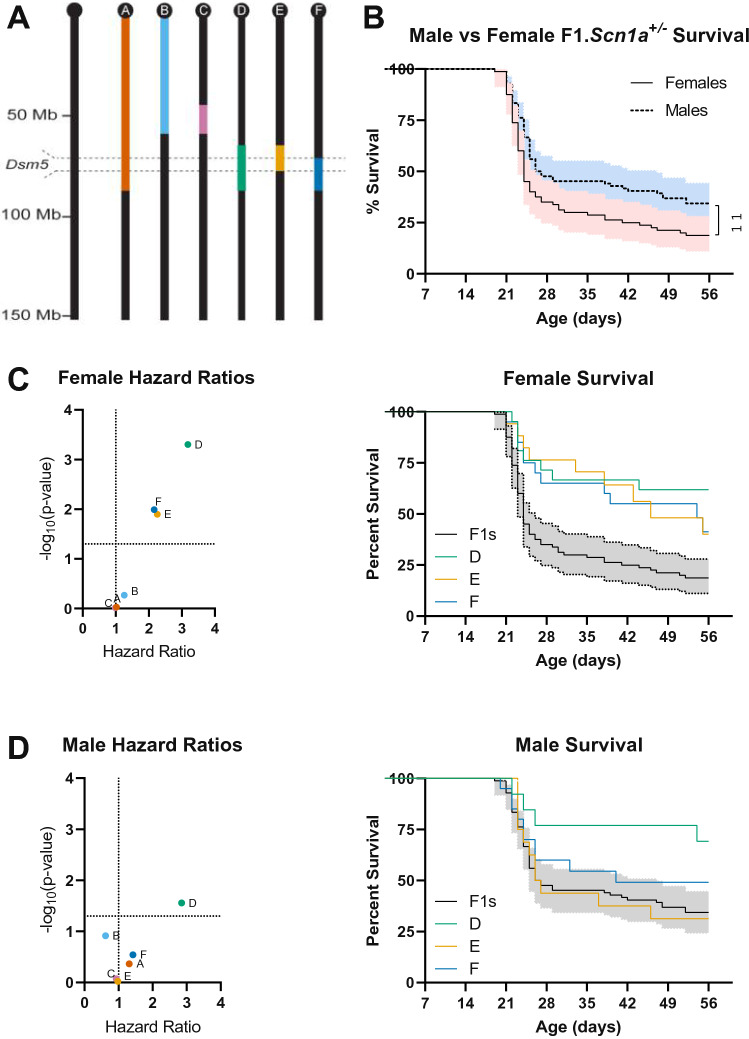
Fine mapping of *Dsm5* with ISC strains. **A** *Dsm5* ISC lines carry varying 129-derived chromosome 11 segments (colors) on a congenic B6 background (black). B6.129-*Dsm5* ISC lines were crossed with 129.*Scn1a*^+/−^ mice and survival of resulting *Scn1a*^+/−^ offspring was monitored to 8 weeks of age. **B** Comparison of female and male F1 controls with heterozygous 129/B6 alleles in *Dsm5* showed a sex-dependent difference in survival, with females (*n* = 80) having worse survival than males (*n* = 84) (*p* < 0.01, LogRank Mantel-Cox). **C–D** Hazard ratios for all ISC lines relative to F1 controls are plotted against –log_10_(p-values) as determined by LogRank Mantel–Cox test, and Kaplan–Meier survival plots are shown for lines ISC-D, ISC-E and ISC-F. Survival was significantly improved in female mice with homozygous 129/129 alleles in ISC-D (*p* < 0.001), ISC-E (*p* < 0.05), and ISC-F (*p* < 0.05) compared to F1.KO heterozygous controls (**C**), while only males with homozygous 129/129 alleles in ISC-D (*p* < 0.05) had improved survival compared to F1 controls (**D**). Shaded areas on Kaplan–Meier plots are 95% confidence intervals for F1 controls with heterozygous 129/B6 alleles

Low resolution mapping of *Dsm5* on chromosome 11 localized a 1.5-LOD support interval to 4.7–39.7 cM (6.99–63.9 Mb; GRCm38.p6) (Miller et al. 2014). To refine the map interval, we used ISC lines carrying varying 129-derived chromosome 11 segments on a congenic B6 background (Fig. 1A; Table 1). Each B6.129-*Dsm5* strain was crossed to 129.*Scn1a*^+/−^ to generate offspring with either homozygous 129/129 or heterozygous 129/B6 alleles in *Dsm5*. Survival of *Scn1a*^+/−^ offspring was monitored to 8 weeks of age and compared between those carrying homozygous (129/129) alleles at *Dsm5* or F1 controls with heterozygous (B6/129) alleles at *Dsm5*. First, we compared survival between males and females with control heterozygous alleles and observed a sex-difference (*p* < 0.01 LogRank Mantel-Cox), with females having worse survival than males (Hazard Ratio (HR): 1.561; 95% confidence interval: 1.088–2.240) (Fig. 1B), consistent with other reports (Gerbatin et al. 2021; Niibori et al. 2020). Therefore, all subsequent ISC comparisons were performed separately by sex. Next, we compared survival between mice that were homozygous 129/129 in each ISC interval with their sex-matched F1 (129/B6) controls. Improvement in survival was observed with some ISC lines (Fig. 1C, D). Improved survival of females was observed with 129/129 alleles from lines ISC-D (65–88 Mb), ISC-E (65–76 Mb) and ISC-F (70–88 Mb) (Fig. 1C), while males only showed improvement in survival with ISC-D. Furthermore, the magnitude of the effect of 129/129 alleles from lines ISC-D was greater in females (HR: 3.183; Table 1) relative to males (HR: 2.851; Table 1) (Fig. 1). This suggests that the effect of 129 alleles in *Dsm5* is more pronounced in females. Under a single modifier gene model, the smallest 129-derived region to harbor a modifier gene would be the ~ 7 Mb region of overlap between strains ISC-E and ISC-F.

**Table 1:** Interval specific mapping

		Females (F1 median survival = 24)			Males (F1 median survival = 27.5)		
Line	Interval	Median Survival (days)	LogRank *p*-value	Hazard Ratio (95% CI)	Median Survival (days)	LogRank *p*-value	Hazard Ratio (95% CI)
A	1 – 88.8 Mb	25.5	0.9999	1.000 (0.5374 to 0.861)	> 56	0.5600	1.217 (0.6454 to 2.295)
B	1—59.3 Mb	25.0	0.4965	1.282 (0.6445 to 2.550)	24	0.0757	0.5840 (0.2671 to 1.277)
C	45.4 – 59.3 Mb	25	0.8791	1.039 (0.6183 to 1.745)	24	0.6925	0.8693 (0.4034 to 1.873)
D	65.4 – 88.4 Mb	** > 56**	**0.0003**	**3.287 (2.073 to 5.210)**	** > 56**	**0.0428**	**2.615 (1.347 to 5.075)**
E	65.4 – 76.1 Mb	**55**	**0.0048**	**2.444 (1.505 to 3.970)**	26.5	0.7614	0.9110 (0.4755 to 1.745)
F	71.8 – 88.4 Mb	**54.5**	**0.0050**	**2.259 (1.422 to 3.588)**	48	0.3604	1.360 (0.7402 to 2.497)

To further refine the ISC intervals, breakpoints were defined by SNP genotyping using the mini MUGA platform that includes over 11,000 SNP probes. For ISC-D and ISC-E, the proximal breakpoint was localized between rs216826621 (65.018062 Mb) and rs48061329 (65.423779 Mb), while the distal breakpoint for ISC-E was between rs26888826 (74.871795 Mb) and rs29407735 (76.169847 Mb). For ISC-D and ISC-F, the distal breakpoint was localized between UNC20075505 (88.402458 Mb) and S3J113539515 (88.487894 Mb), while the proximal breakpoint for ISC-F was between gUNC19880825 (70.808812 Mb) and rs26890640 (71.870339 Mb). Thus, the area of overlap between ISC-E and ISC-F is defined as the 5.4 Mb region between 70.808812 Mb and 76.169847 Mb. Bold indicates *P* < 0.05.

### Candidate gene analysis

Under a single modifier gene model, the *Dsm5* locus was narrowed to a 5.4 Mb interval (70.808812–76.169847 Mb, GRCm38.p6) that contains 126 known genes, including 12 RNA genes, and 114 protein coding genes (including 30 olfactory receptors genes). Of those, 28 protein coding and 10 RNA genes have confirmed brain expression.

**Fig. 2:**
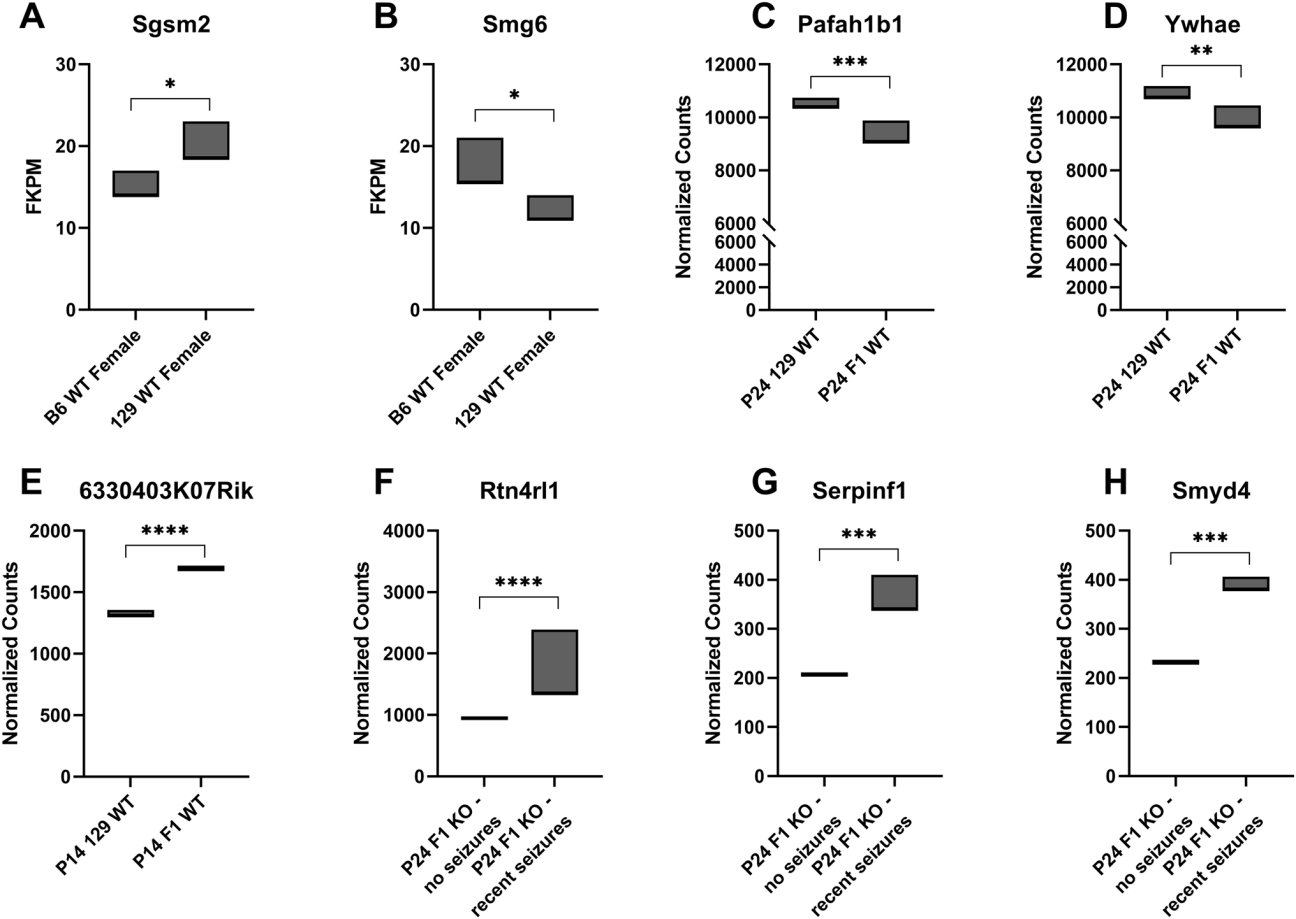
Differential expression of positional candidate modifier genes from our published RNA-seq datasets (Hawkins et al. 2019, 2016). **A–B** Two genes, *Sgsm2* (**A**) and *Smg6* (**B**) had differential expression between 129 and B6 females (Hawkins et al. 2016). **C–E** Three genes were differentially expressed between wild-type 129 and F1 mice in pooled samples containing both sexes, with *Pafah1b1* (**C**) and *Ywhae* (**D**) differentially expressed at P24*, *and *6330403K07Rik* differentially expressed at P14 (**E**) (Hawkins et al. 2019). **F–H** Comparison of F1.*Scn1a*^+/−^ mice with or without seizures in the antecedent 24 h showed differential expression of three genes, *Rtn4rl1* (**F**), *Serpinf1* (**G**) and *Smyd4* (**H**) (Hawkins et al. 2019). Floating bars represent the range of values for each group and significance is denoted as *p < 0.05, **p < 0.01, ***p < 0.001, ****p < 0.0001

**Fig. 3:**
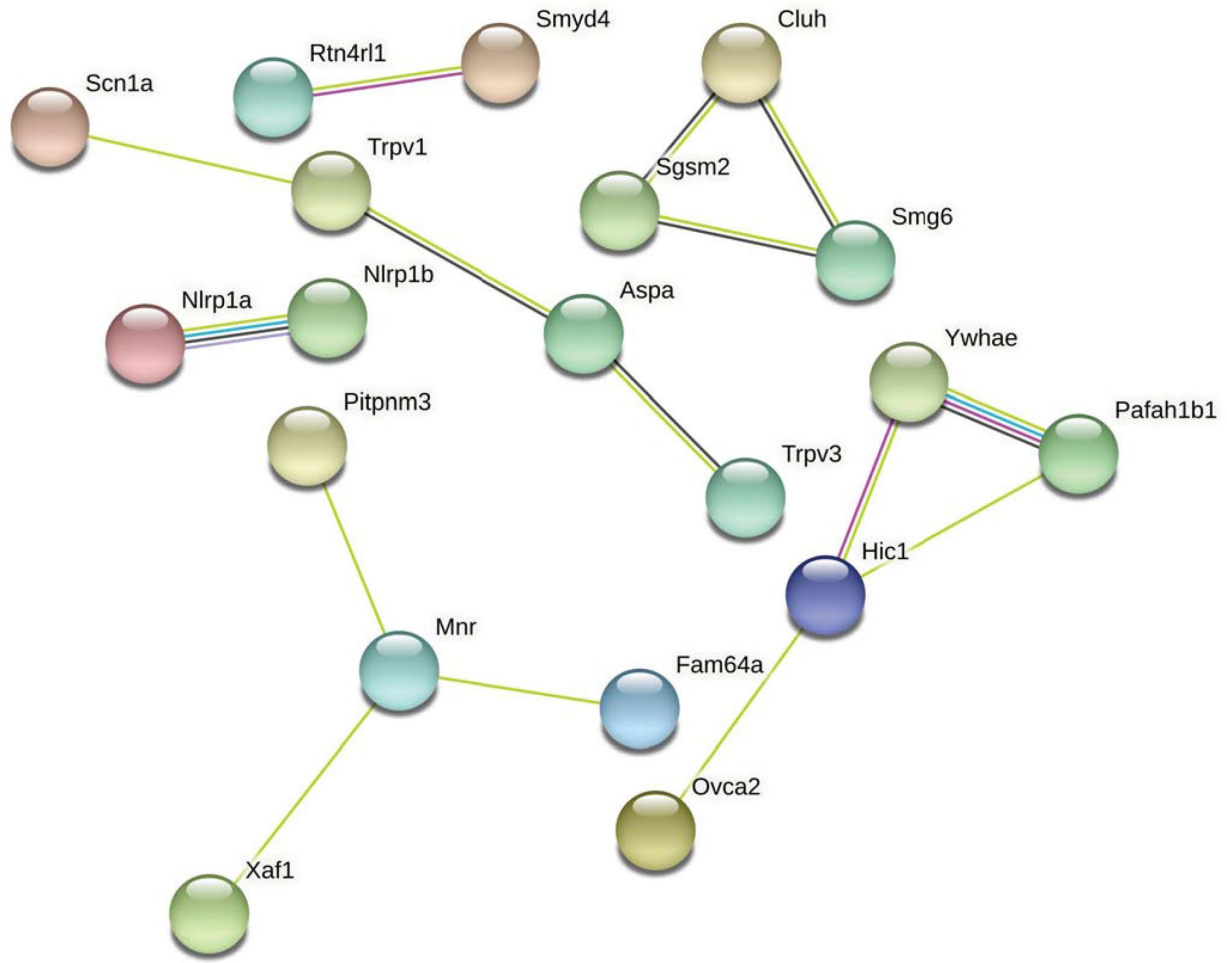
String protein association network for candidate protein coding modifier genes and *Scn1a*. Only *Trpv1* had a direct association with *Scn1a*, while *Aspa* and *Trpv3* had indirect associations. The 30 protein coding candidate genes in Table 2 and *Scn1a* were used as input in String (v. 11.0) (Szklarczyk et al. 2019). At least medium confidence associations are displayed (interaction score ≥ 0.4). Edges represent protein associations, with line color indicating the type of evidence as follows: yellow, text-mining; black, co-expression; pink, experimentally determined interactions; blue, known interactions from curated databases; purple, protein homology

Examination of candidate genes within the refined *Dsm5* interval in existing mRNA-seq datasets revealed two differentially expressed genes (DEGs) between wild-type 129 and B6 females (*Sgsm2, Smg6*) (Fig. 2A, B); and three DEGs between P14 or P24 wild-type 129 and F1 mice in pooled samples containing both sexes (*Pafah1b1, Ywhae, 6330403K07Rik*) (Fig. 2C-E). There were three DEGs (*Rtn4rl1,* *Serpinf1, Smyd4*) when comparing F1.*Scn1a*^+/−^ mice without seizures and F1.*Scn1a*^+/−^ mice with recent seizures (Fig. 2F-H). From whole genome sequencing, we identified 24 genes in the refined *Dsm5* interval with predicted deleterious variants, including missense, splice site, and indels (*4930563E22Rik, 4933427D14Rik, 6330403K07Rik, Aspa, Cluh, E130309D14Rik, Pimreg, Haspin, Hic1, Itgae, Nlrp1a, Nlrp1b, Ovca2, Pitpnm3, Rpa1, Rtn4rl1, Smg6, Spns3, Tax1bp3, Tekt1, Trpv1, Trpv3, Xaf1, Zzef1*) (Table 2). Of the 30 protein coding genes with identified coding sequence or expression differences, ten had a prior association with seizure or epilepsy based on literature and database searches, including *Aspa, Hic1, Nlrp1a, Nlrp1b, Pafah1b1, Smg6, Trpv1, Trpv3, Ywhae* and *6330403K07Rik* (Table 2). Pathway analysis of these *Dsm5* candidate genes showed that *Trpv1* is the only first neighbor to *Scn1a*, while *Trpv3* and *Aspa* are included in this network through their interactions with *Trpv1* (Fig. 3).

**Table 2:** Single-gene model positional candidate protein coding genes with expression and/or coding differences between B6 and 129

Gene	NCBI Gene ID	Chromosome 11 Location (GRCm38.p6)	Expression difference	Coding difference	Prior association with epilepsy and/or Seizures
*6330403K07Rik*	103,712	71,031,941–71,033,513	WT 129 v WT F1	Yes	Yes
*Nlrp1a*	195,046	71,092,236–71,144,704		Yes	Yes
*Nlrp1b*	637,515	71,153,102–71,230,733		Yes	Yes
*Pimreg*	109,212	72,042,032–72,047,370		Yes	
*Pitpnm3*	327,958	72,047,528–72,135,778		Yes	
*4933427D14Rik*	74,477	72,153,929–72,207,459		Yes	
*4930563E22Rik*	75,304	72,215,138–72,218,444		Yes	
*Xaf1*	327,959	72,301,629–72,313,733		Yes	
*Tekt1*	21,689	72,344,722–72,362,442		Yes	
*Spns3*	77,577	72,494,919–72,550,506		Yes	
*Zzef1*	195,018	72,796,226–72,927,120		Yes	
*Itgae*	16,407	73,090,583–73,147,446		Yes	
*Haspin*	14,841	73,135,485–73,138,294		Yes	
*Tax1bp3*	76,281	73,177,083–73,183,162		Yes	
*Trpv1*	193,034	73,234,292–73,261,242		Yes	Yes
*Trpv3*	246,788	73,267,388–73,300,363		Yes	Yes
*Aspa*	11,484	73,304,992–73,329,596		Yes	Yes
*E130309D14Rik*	432,582	74,619,605–74,641,516		Yes	
*Cluh*	74,148	74,649,495–74,670,847		Yes	
*Pafah1b1*	18,472	74,673,949–74,724,670	WT 129 vs WT F1		Yes
*Sgsm2*	97,761	74,849,261–74,897,060	WT 129 vs WT B6 (Females)		
*Smg6*	103,677	74,925,823–75,164,448	WT 129 vs WT B6 (Females)	Yes	Yes
*Hic1*	15,248	75,164,565–75,169,519		Yes	Yes
*Ovca2*	246,257	75,175,942–75,178,835		Yes	
*Rtn4rl1*	237,847	75,193,783–75,267,769	F1 KO v F1 KO with recent seizures	Yes	
*Rpa1*	68,275	75,298,166–75,348,324		Yes	
*Smyd4*	319,822	75,348,433–75,405,705	F1 KO v F1 KO with recent seizures		
*Serpinf1*	20,317	75,409,769–75,422,701	F1 KO v F1 KO with recent seizures		
*Ywhae*	22,627	75,732,869–75,765,845	WT 129 vs WT F1		Yes

**Fig. 4:**
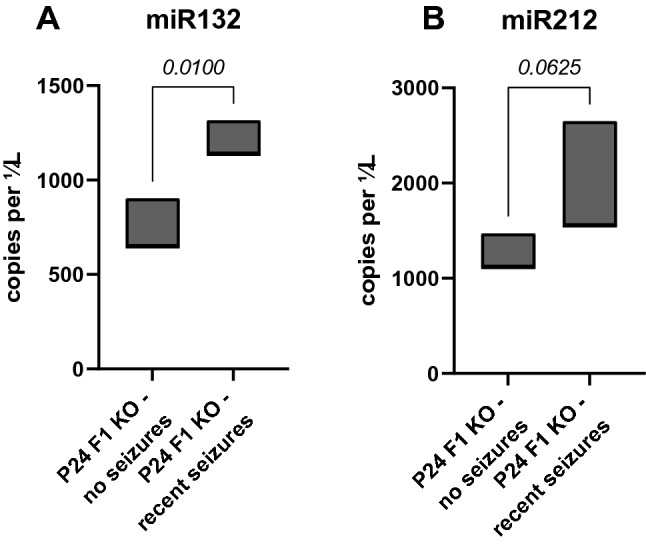
Seizure responsive positional candidate miRNA genes with seed match sites in the *Scn1a* 3’UTR. F1.*Scn1a*^+/−^ mice with recent seizures had elevated levels of miR-132-3p (**A**) and a trend toward elevated levels of miR-212-3p (**B**) relative to those that were seizure-free in the antecedent 24 h. Each group had three pooled samples consisting of 4 mice each, with either no seizures or 3–7 seizures per mouse in the 24 h prior to collection. Floating bars represent the range of values for each group. *p*-values are from Welch’s *t*-tests

Three of the brain-expressed miRNAs (miRs-22, -132, -212) had prior association as seizure-responsive genes according to EpimiRBase, a database cataloging published reports of miRNA up- and downregulation following seizures (Jimenez-Mateos et al. 2011; Mooney et al. 2016). It is notable that two of these miRNAs (miRs-132, and -212) are shown to be upregulated following seizures and are predicted to target voltage-gated sodium channel genes, including *Scn1a*. Furthermore, a recent study demonstrated that miR-132 regulates Nav1.1 expression in a chronic cerebral hypoperfusion model (Hu et al. 2019). To assess whether seizures affected expression of miR-132-3p or miR-212-3p, we performed RT-ddPCR and compared expression between F1.*Scn1a*^+/−^ mice with and without recent seizures. Consistent with prior reports, we found that F1.*Scn1a*^+/−^ mice with recent seizures had elevated levels of miR-132-3p and a trend toward elevated levels of miR-212-3p relative to those that were seizure-free in the antecedent 24 h (Fig. 4). This suggests the possibility that seizure-mediated elevation of miR-132-3p and/or miR-212-3p could downregulate expression of Na_V_1.1 and exacerbate the effect of *Scn1a* heterozygous deletion.

## Discussion

In the present study, we constructed a set of ISCs to fine map the *Dsm5* locus on mouse chromosome 11. Under a single gene modifier model, our results narrow the modifier interval to a 5.7 Mb region of overlap between ISC-E and ISC-F. Within this interval, we identified a number of potential candidate modifier genes with coding sequence variation and/or expression differences between the strains. However, future studies will be needed to validate candidate modifier genes.

Among the *Dsm5* potential modifier genes, *Trpv1* is an intriguing candidate as a proposed target of cannabidiol, an FDA/EMA-approved Dravet syndrome therapeutic (Gray and Whalley 2020). Recently, we reported differential expression of *Trpv1* transcript in cortex, with higher expression in F1.*Scn1a*^+*/−*^ mice relative to seizure resistant 129.*Scn1a*^+*/−*^ mice (Satpute Janve et al. 2021). However, deletion of *Trpv1* had both pro- and anti-convulsant effects when combined with the *Scn1a*^+/−^ allele. Double mutant *F1.Scn1a*^+*/−*^*;Trpv1*^+*/−*^ mice had lower temperature thresholds for hyperthermia-induced seizure compared to F1.*Scn1a*^+/−^ mice, suggesting a pro-convulsant effect. Conversely, *Trpv1* deletion resulted in reduced severity of spontaneous GTCS in double mutant F1*.Scn1a*^+*/−*^*;Trpv1*^+*/−*^ mice, although it did not affect seizure frequency or survival compared to F1.*Scn1a*^+/−^ mice (Satpute Janve et al. 2021). Although *Trpv1* deletion alters seizure phenotypes in *Scn1a*^+/−^ mice, it is not possible to separate an effect of *Trpv1* deletion from residual 129 alleles in the chromosome 11 region remaining from the homologous recombination in JM-1 ES cells (129X1/SvJ) (Caterina et al. 2000). Lack of any effect of a Trpv1-selective antagonist on F1.*Scn1a*^+/−^ phenotypes lends support for an effect of residual 129 alleles rather than *Trpv1* deletion itself on *Scn1a*^+/−^ seizure phenotypes (Satpute Janve et al. 2021). Additional intriguing candidates include the miRNA genes for miR-132-3p and miR-212-3p that are predicted to target *Scn1a* and are seizure-responsive genes. However, to date, we have no evidence for altered expression of *Scn1a* transcript between strains or in response to seizures. It is possible that miRNA regulation could be post-transcriptional and alter protein levels,

The *Dsm5* region overlaps previously identified seizure susceptibility QTL, including *Szs10* and *Gss4* (Ferraro et al. 2001; Gu et al. 2020). In addition, this region of mouse chromosome 11 is syntenic with recurrent CNVs implicated in epilepsy and neurodevelopmental disorders (Coppola et al. 2019; Kolishovski et al. 2019). This includes synteny with Miller-Dieker lissencephaly 17p13.3 microdeletion syndrome, which includes epilepsy as part of the neurological features. The 17p13.3 microdeletion includes deletion of *PAFAH1B1* and *YWHAE*, as well as deletion of the intervening genes. The lissencephaly phenotype is attributed to deletion of *PAFAH1B1*, formerly known as *LIS1*, while disruption of *YWHAE*, encoding 14–3-3ε, is associated with variable structure brain abnormalities, cognitive impairment and seizures (Cardoso et al. 2000; Noor et al. 2018; Romano et al. 2020). Rare microduplications at 17q12 are associated with epilepsy, including familial FS/GEFS + that is also often caused by *SCN1A* variants (Hardies et al. 2013; Mefford et al. 2016, 2007). Genes associated with the 17q12 microduplication are *AATF, ACACA, C17orf78, DDX52, DHRS11, DUSP14, GGNBP2, HNF1B, LHX1, MRM1, MYO19, PIGW, SYNRG, TADA2A*, and *ZNHIT3* (Mefford et al. 2016). *LHX1*, encoding LIM homeobox protein 1, and *ACACA*, encoding acetyl-CoA carboxylase, have been proposed as potential candidate genes responsible for neurodevelopmental and epilepsy phenotypes, although there is not sufficient evidence to confirm causality to date (Hardies et al. 2013; Mefford et al. 2007). The 17p13.3 deletion overlaps with the region of overlap between ISC-E and ISC-F, while the 17q12 microduplication overlaps with the non-overlapping segment of ISC-F.

One caveat of our study is that the fine mapping data could also support an alternative interpretation of additive modifier genes from the non-overlapping portions of ISC-E and ISC-F. This seems less likely when examining the Kaplan–Meier survival curves (Fig. 1C, D), where the net effect of ISC-E, ISC-F or ISC-D appear similar and are not significantly different from one another (*p* > 0.3, LogRank Mantel-Cox). However, examination of the female hazard ratios shows a modest trend toward smaller magnitude of effects for ISC-E (2.258 HR) and ISC-F (2.162 HR) relative to ISC-D (3.183 HR) (Fig. 1C), which could support the possibility of each interval harboring modifiers with additive effects. Additional support for this hypothesis comes from males that showed robust improvement in survival with ISC-D, whereas ISC-E or ISC-F alone had no protective effect. Ideally, these two possibilities could be tested empirically by generation of ISC lines separating the E–F overlapping and non-overlapping regions; however, such recombination events have been elusive in our ISC colony. It may be possible in the future to use genome engineering to further dissect the interval.

Haploinsufficiency for *SCN1A* is a major cause of Dravet syndrome, and yet there is variable expressivity among patients with this shared genetic basis. This suggests that clinical presentation is influenced by other factors, which may include genetic modifiers. Identification of modifier genes that influence disease course will provide insights into understanding the molecular basis of Dravet syndrome and identify potential pathways for intervention.

## Data Availability

Genomic and transcriptomic datasets generated during and/or analyzed during the current study are available in the NCBI GEO repository [GSE112627] and SRA repository [PRJNA817075]. Other datasets are available from the corresponding author on reasonable request.
